# Cellulose nanocrystals from agriculture and forestry biomass: synthesis methods, characterization and industrial applications

**DOI:** 10.1007/s11356-024-35127-3

**Published:** 2024-09-28

**Authors:** Sundus Saeed Qureshi, Sabzoi Nizamuddin, Jia Xu, Tony Vancov, Chengrong Chen

**Affiliations:** 1 Australian Rivers Institute and School of Environment and Science, Griffith University, Nathan Campus, Brisbane, Queensland 4111 Australia; 2 Cooperative Research Centre for High Performance Soils, Callaghan, NSW Australia; 3 Water Regulation Division, Grampians Wimmera Mallee Water (GWMWater) Corporation, Horsham, Victoria 3400 Australia; 4 NSW Department of Primary Industries, Elizabeth Macarthur Agricultural Institute, Menangle, NSW 2568 Australia

**Keywords:** Cellulose nanocrystals, Agricultural biomass resources, Hydrolysis, Delignification, Industrial applications, Synthesis methods

## Abstract

Agricultural and forestry biomass wastes, often discarded or burned without adequate management, lead to significant environmental harm. However, cellulose nanocrystals (CNCs), derived from such biomass, have emerged as highly promising materials due to their unique properties, including high tensile strength, large surface area, biocompatibility, and renewability. This review provides a detailed analysis of the lignocellulosic composition, as well as the elemental and proximate analysis of different biomass sources. These assessments help determine the yield and characteristics of CNCs. Detailed discussion of CNC synthesis methods -ranging from biomass pretreatment to hydrolysis techniques such as acid, mineral, solid acid, ionic liquid, and enzymatic methods—are provided. The key physical, chemical, and thermal properties of CNCs are also highlighted, particularly in relation to their industrial applications. Recommendations for future research emphasize the need to optimize CNC synthesis processes, identify suitable biomass feedstocks, and explore new industrial applications.

**Graphical Abstract:**

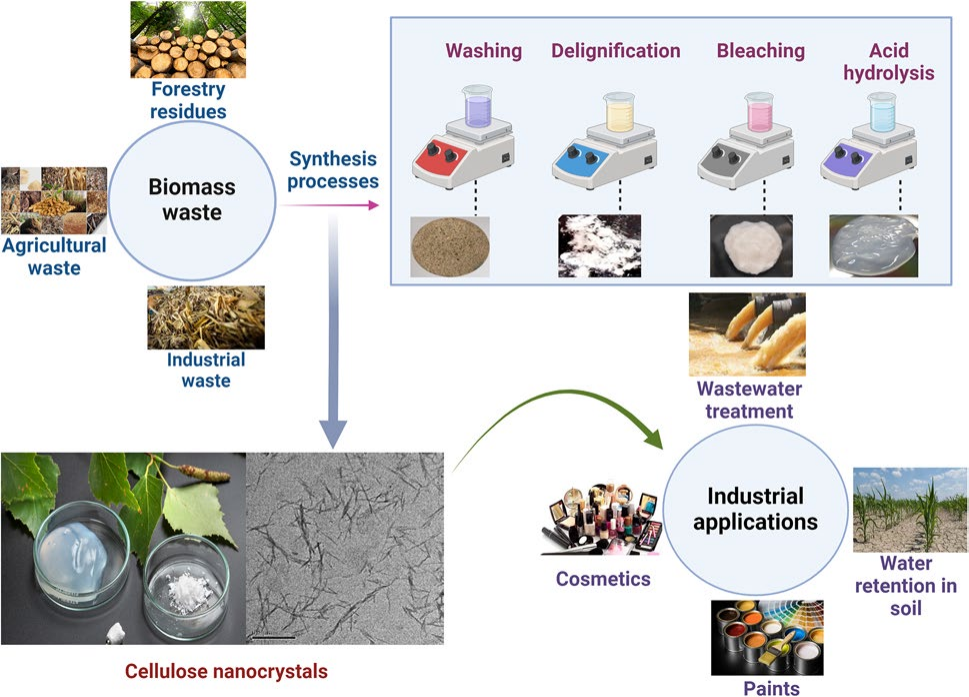

**Supplementary Information:**

The online version contains supplementary material available at 10.1007/s11356-024-35127-3.

## Introduction

The continuous rise in global energy consumption, driven by population growth and industrialization, has led to environmental issues such as global warming and pollution (Gutiérrez et al. 2020). This is largely due to the heavy reliance on non-renewable resources like fossil fuels and plastics, which contribute significantly to environmental degradation. Therefore, it is important to look for alternate options of petroleum-based fuels and plastics. In response, there has been a growing shift towards renewable and biodegradable materials that can reduce carbon emissions and environmental waste. Biomass, derived from agricultural and forestry residues, is a renewable resource with considerable potential as an eco-friendly alternative. It is abundant, biodegradable, and offers a lower environmental footprint compared to traditional materials. Of particular interest is cellulose, a key component of biomass, which can be converted into CNCs. CNCs are gaining recognition for their potential applications across various industries, including packaging, biomedicine, and electronics. Unlocking the full potential of CNCs requires a detailed understanding of biomass composition and the optimization of extraction processes.

Using renewable, eco-friendly resources and developing value-added products from biomass present promising solutions to these environmental challenges. Biomass from biological sources contains multiple components, including cellulose, hemicellulose, and lignin, along with extractives, fats, proteins, sugars, moisture, and ash. The potential of biomass in biorefining processes aimed at producing chemicals, biofuels, and other valuable materials is still underexplored (Wang et al. 2021). Typically, plant biomass contains 35–50% cellulose, 20–35% hemicellulose, 15–20% lignin and 15–20% of fats, ash, extractives and proteins (Nizamuddin et al. 2018). Globally, the total annual biomass production from agriculture and forestry is estimated at approximately 11.9 billion tons of dry matter, with agriculture contributing 61% and forestry 39% (Popp et al. 2021).

Agricultural and forestry wastes, such as crop residues (corn stover, wheat and rice straw, maize, sunflower) and woody biomass, offer significant environmental benefits due to their renewable nature, sustainability, and low production costs. These materials are often rich in cellulosic content, which can be isolated through various chemical and physical methods (Reddy et al. 2016). Previous research has demonstrated the strong potential of these waste materials as feedstock for biorefining processes, enabling the production of chemicals, biofuels, and other high-value products.

Nanocellulose, a derivative of cellulose, exhibits nanoscale dimensions and shares properties like high strength and hydrophilicity with its parent material (Thomas et al. 2020b). Its properties, including shape, size, and functionality, can vary depending on the synthesis method and the composition of the raw material. Among its various forms, CNCs—also known as cellulose nano-whiskers (Motta Neves et al. 2020) or crystalline nanocellulose (Lenfant et al. 2017)—are rod-like with diameters ranging from 2–20 nm and lengths between 100–500 nm. These crystals feature a large surface area (~ 150 m^2^/g), a high crystallinity index (> 70%), exceptional tensile strength (7500 MPa), and a high aspect ratio (~ 70) (Tang et al. 2014). These properties make CNCs suitable for a wide range of industrial applications, including healthcare (Favatela et al. 2021; Thomas et al. 2020a), electronics (Nyamayaro et al. 2020; Yao et al. 2020), construction (Ghahari et al. 2020; Lee et al. 2019), food packaging (Alvarado et al. 2018; He et al. 2021), 3D printing inks (Dai et al. 2019; Li et al. 2021; Wang et al. 2018), oilfield servicing fluids (Li et al. 2015, 2021), and polymer composites (Kargarzadeh et al. 2018).

This review provides an in-depth discussion of CNC production methods from agricultural and forestry biomass, examining the chemical and lignocellulosic composition of various feedstocks. It explores the synthesis of CNCs from agricultural biomass, highlighting the pre-treatment steps and different types of hydrolysis techniques. Additionally, the chemical, thermal, and structural properties of various types of CNCs are examined, alongside their diverse industrial applications.

## Agricultural and forestry biomass

The expansion of agricultural and forestry activities has led to a significant increase in biomass residues (Anwar et al. 2021b), which hold potential as valuable resources for producing value-added products. When left unmanaged, these residues contribute to resource wastage and environmental challenges. However, converting them into value-added products like CNCs can promote resource efficiency and reduce environmental impact (Yu et al. 2023).

Agricultural and forestry biomass are rich in lignocellulosic materials, and the composition of these materials plays a critical role in determining the yield and quality of CNCs. Factors such as the source, growth conditions, and age of the biomass significantly affect its lignocellulosic structure and elemental properties, which in turn influence CNC yield and quality (Nizamuddin et al. 2016b). Studies have confirmed the importance of lignocellulosic composition in assessing the suitability of biomass for CNC production (Nizamuddin et al. 2016a). Additionally, elemental and proximate analyses are essential for understanding the potential yield and quality of CNCs.

Proximate analysis, following the ASTM D3173-75 standard, evaluates key biomass properties, including moisture content, ash content, volatile matter, and fixed carbon (FC) (Xaba et al. 2022). For example, CNC films derived from corncob, corn husk, wheat bran, and coconut shell exhibit moisture contents of 18.3%, 20%, 20.9%, and 15.1%, respectively. Higher solid content in CNCs from corncob and coconut shell suggests a greater presence of water-insoluble particles and non-volatile substances (de Andrade et al. 2019). Similarly, elemental analysis, also known as ultimate analysis, determines the percentages of nitrogen (N), hydrogen (H), carbon (C), oxygen (O), and sulfur (S) in biomass, as outlined by ASTM D3174-76 (Popoola et al. 2019).

Attia et al. (2019) found that carbon content decreases during fabrication, from bagasse to CNC, due to the removal of volatile matter, oxygen, and hydrogen during carbonization. Conversely, CNCs retain higher percentages of nitrogen, oxygen, and hydrogen due to acid hydrolysis and thermal stabilization, which enhance hydrogen bonding between cellulose hydroxyl (OH) groups (Zhao et al. 2009). Similar findings were reported in CNCs produced from jackfruit peel, which contained 42.2% carbon, 1.5% nitrogen, and 6.7% hydrogen (Trilokesh and Uppuluri 2019). Table 1 outlines the proximate and ultimate analyses of various biomass types, providing valuable insights into their suitability for CNC production.

**Table 1:** Ultimate and proximate analysis of some biomass

	Biomass	FC (%)	VM (%)	Ash (%)	C (%)	H (%)	O (%)	References
Agricultural by-products	Coconut coir	29.7	66.58	3.72	50.29	5.05	39.63	(Demirbaş 1997)
	Corn cob	15.8	70.24	7.5	44.53	6.89	45.97	(Cuiping et al. 2004)
	Alfalfa Stalk	15.62	68.02	5.84	40.60	5.15	36.02	(Tillman 2000)
	Sugarcane bagasse	15.0	-	11.3	44.8	5.4	39.6	(Demirbas 2004, Ebeling and Jenkins 1985)
	Olive Pitts	21.2	75.6	3.2	48.81	5.79	43.48	(Parikh et al. 2007)
	Urban wood waste	12.5	52.56	4.08	33.22	3.84	27.04	(Tillman 2000)
	Olive cake pellets	15.7	64.2	8.2	42.1	4.99	31	(Khan et al. 2009)
	Willow wood	16.07	82.22	1.71	49.90	5.90	41.80	(Jenkins et al. 1998)
	Oil palm shell	15.75	77.91	6.34	51.1	6	42.9	(Hasegawa et al. 2004)
	Sawdust	9.34	55.03	0.69	32.06	3.86	27.04	(Tillman 2000)
	Akhrot shell	18.78	79.98	1.2	49.81	5.64	42.94	(Channiwala 1992, Channiwala and Parikh 2002)
	Almond hulls	20.07	73.8	6.13	47.53	5.97	39.16	(Miles et al. 1995)
	Rice husk	16.95	61.81	21.24	38.5	5.2	34.61	(Channiwala and Parikh 2002)
	Almond shells	20.71	76	3.29	49.3	5.97	40.63	(Demirbas 2004, 2005, Miles et al. 1995)
	Sal seed husk	28.06	62.54	9.4	48.12	6.55	35.93	(Channiwala and Parikh 2002)
	Wood pallets (pine)	14.5	80.4	0.2	45.5	6.6	47.7	(Khan et al. 2009)
	Olive husk	18.4	77.5	4.1	49.9	6.2	42.0	(Demirbas 2004)
	Green house residue	5.5	61	31	47.1	7.4	10.9	(Khan et al. 2009)
	Sunflower pellets	19.5	65.2	4.1	44.1	5.17	34.6	(Khan et al. 2009)
	Coconut shell	22.1	77.19	0.71	50.22	5.7	43.37	(Channiwala 1992, Channiwala and Parikh 2002)
	Coconut shell	22.1	77.19	0.71	50.22	5.7	43.37	(Channiwala 1992, Channiwala and Parikh 2002)
	Pistachio shell	16.84	82.03	1.13	48.79	5.91	43.41	(Parikh et al. 2007)
	Groundnut shell	21.6	72.7	5.7	48.59	5.64	39.49	(Channiwala &Parikh 2002, Parikh et al. 2007)
	Peanut hull	21.09	73.02	5.89	45.77	5.46	39.56	(Parikh et al. 2007)
	Brazil nut shell	22.2	76.1	1.7	49.15	5.7	42.8	(Bonelli et al. 2001)
	Hazelnut shell	28.3	69.3	1.4	52.9	5.6	42.7	(Demirbas 2004, Parikh et al. 2007)
	Chapparal wood	18.68	75.19	6.13	46.9	5.08	40.17	(Channiwala and Parikh 2002)
	Pinewood	10.3	82.4	1.5	49	6.4	44.4	(Naik et al. 2010)
	Beech wood	24.6	74	0.4	49.5	6.2	41.2	(Demirbaş 1997)
	Timothy grass	16	77.9	1.1	42.2	6	50.4	(Naik et al. 2010)
	Ailanthus wood	24.8	73.5	1.7	49.5	6.2	41	(Demirbaş 1997)
	Bamboo wood	11.24	86.8	1.95	48.76	6.32	42.77	(Channiwala and Parikh 2002)
	Flax straw	8.8	80.3	3	43.1	6.2	49.9	(Naik et al. 2010)
	Red wood	19.92	79.72	0.36	50.64	5.98	42.88	(Boocock and Sherman 1985)
	Barley straw	4.8	78.5	9.8	41.4	6.2	51.7	(Naik et al. 2010)
	Douglas fir wood	12.6	87.3	0.1	50.64	6.18	43	(Channiwala and Parikh 2002)
	Red oak wood	21.5	78.6	0.5	50	6	42.4	(Demirbaş 2004)
	Casurina wood	19.58	78.58	1.83	48.5	6.24	43.12	(Channiwala 1992)
	Coal	43.6	2.4	8.3	81.5	4	3.3	(Demirbaş 2003)
	Neem wood	12.19	85.86	1.93	48.26	6.27	43.46	(Channiwala and Parikh 2002)
	Pine tree	14.45	76.5	0.89	49.41	7.67	42.19	(Cuiping et al. 2004)
	Spruce wood	29.3	70.2	1.5	51.9	6.1	40.9	(Demirbaş 1997)
	Rice straw	15.55	61.1	15.25	38.52	6.13	38.28	(Cuiping et al. 2004)
	Sunflower shell	19.8	76.2	4	47.4	5.8	41.3	(Demirbas 2004)
	Subabul wood	18.52	81.02	1.2	48.15	5.87	44.75	(Channiwala 1992)
	Peanut stalk	15.66	66.67	9.12	40.28	7.18	42.47	(Cuiping et al. 2004)
	Cotton stalk	18.57	67.63	6.41	46.1	6.85	43.35	(Cuiping et al. 2004)
	Wood Bark	31.8	66.6	1.6	53.1	6.1	40.6	(Demirbaş 1997)
	Rape stalk	17.26	72.99	3.6	42.42	7.06	46.1	(Cuiping et al. 2004)
	Broad bean stalk	18.9	68.44	5.03	42.16	6.13	45.28	(Cuiping et al. 2004)
	Wood chips	23.5	76.4	0.1	48.1	5.99	45.74	(Parikh et al. 2007)
	Wheat straw	14.96	63.96	12.45	42.11	6.53	40.51	(Cuiping et al. 2004)
	Corn straw	14.83	62.74	13.12	42.69	6.16	42.69	(Cuiping et al. 2004)
	Walnut shell	37.9	59.3	2.8	53.4	6.6	45.4	(Demirbas 2004)
	Coconut fibre	26.6	70.6	2.8	46.43	5.49	43.78	(Demirbaş 1997)
	Soya bean	15.62	68.95	6.08	43.16	6.9	44.76	(Cuiping et al. 2004)
	Tea waste	13	85.5	1.5	48	5.5	44	(Demirbas 2004)
	Sesame stalk	17.3	68.93	6.11	41.34	6.57	45.16	(Cuiping et al. 2004)
	Mulberry stick	22.8	75.1	2.1	44.23	6.61	46.25	(Parikh et al. 2005)
	Peach pit	19.8	79.1	1.1	49.14	6.34	43.52	(Parikh et al. 2005)
Plant species	Bowdichia nitida	18.2	81.8	0.1	52.3	6.1	41.3	(Telmo et al. 2010)
	Switch grass	12.19	65.19	7.63	39.68	4.95	31.77	(Tillman 2000)
	Salix Babylonica	16.8	80.8	2.4	47.2	5.6	44.4	(Telmo et al. 2010)
	Hymenaea courbaril	17.6	81.7	0.7	48.3	5.7	45.1	(Telmo et al. 2010)
	Prunus avium	15	84.9	0.4	48.6	5.8	45.3	(Telmo et al. 2010)
	Rubber plant	18.3	62.92	9.9	48.69	7.29	39.03	(Cuiping et al. 2004)
	Quercus robur	18	81.7	0.3	47.2	5.5	46.8	(Telmo et al. 2010)
	Sena leaves	25.5	57.2	17.3	36.2	4.72	37.49	(Parikh et al. 2007)
	Fagus sylvatica	13.9	85.7	0.5	46.2	5.8	47.2	(Telmo et al. 2010)
	Pine needles	26.12	72.38	1.5	48.21	6.57	43.72	(Demirbaş 1997)
	Castanea sativa	20.3	79.6	0.1	47.1	4.9	47.7	(Telmo et al. 2010)
	Peanut shuck	16.85	61.64	12.15	45.9	6.74	42.79	(Cuiping et al. 2004)
	Cedrus atlantica	16.7	82.9	0.4	50.3	5.6	43.6	(Telmo et al. 2010)
	Cotton shuck	20.74	62.16	6.88	44.54	6.6	46.66	(Cuiping et al. 2004)
	*Pinus pinaster*	14.1	85.8	0.2	48.4	6	45.3	(Telmo et al. 2010)
	Poplar	15.42	74.04	2.63	47.46	6.74	44.5	(Cuiping et al. 2004)
	Met sequoia	16.11	74.3	2.2	47.98	6.82	43.98	(Cuiping et al. 2004)
	Phoenix tree	18.29	68.68	5.28	48.14	7.88	39.84	(Cuiping et al. 2004)
	Birch tree	13.68	74.91	2.36	48.32	8.36	40.6	(Cuiping et al. 2004)
Environmental wastes	Sewage sludge	7	44.6	41.5	52	6.3	32.1	(Khan et al. 2009)
	Hybrid poplar	12.49	84.81	2.70	50.18	6.06	40.43	(Jenkins et al. 1998)

*VM* Volatile matter, *C* Carbon, *H* Hydrogen, *O* Oxygen

The composition of lignocellulosic biomass varies depending on the plant’s growth conditions and duration. The plant cell wall consists of cellulose, hemicellulose, and lignin, with varying proportions depending on the biomass source (Boro et al. 2022). Table 2 provides an overview of the lignocellulosic composition of various biomass materials. Cellulose, a linear polymer made up of anhydro-glucose subunits linked by β-1,4-glucosidic bonds, forms fibers composed of crystalline domains separated by amorphous regions (Mukherjee et al. 2019). These cellulose fibers, embedded in a matrix of hemicellulose and lignin, provide structural integrity and resist degradation (Coelho et al. 2024; Shakeel et al. 2024). The crystalline regions possess a highly ordered and rigid structure, making them resistant to acid hydrolysis, while the amorphous regions are more susceptible to chemical breakdown (Haldar & Purkait 2020). Intramolecular hydrogen bonds bind microfibrils together, forming the cellulose fibers that provide structural integrity.

**Table 2:** Main components of some biomass

	Biomass	Cellulose (%)	Hemicellulose (%)	Lignin (%)	References
Agricultural by-products	Banana stem	37.92	71.15	12.25	(Minowa et al. 1998)
	Bamboo	47	25	21	(Ando et al. 2000)
	Corn Stover	51.2	30.7	14.4	(Demirbaş 1997)
	Rubber tree	45.84	73.84	21.42	(Minowa et al. 1998)
	Tobacco stalk	42.4	28.2	27	(Demirbaş 1997)
	Rice straw	36.26	74.45	34.73	(Minowa et al. 1998)
	Tobacco leaf	36.3	34.4	12.1	(Demirbaş 1997)
	Rice husk	44.14	82.42	42.58	(Minowa et al. 1998)
	Ailanthus wood	46.7	26.6	26.2	(Demirbaş 1997)
	Pineapple leaf	32.16	63.2	18.68	(Minowa et al. 1998)
	Softwood	45.8	24.4	28	(Demirbaş 1997)
	Oil palm shell	27.7	21.6	44	(Abnisa et al. 2011)
	Hardwood	45.2	31.3	21.7	(Demirbaş 1997)
	Oil palm petioles	37.01	71.65	20.94	(Minowa et al. 1998)
	Oil palm husk	34.28	61.31	31.91	(Minowa et al. 1998)
	Oil palm EFB	35.71	65.57	21.97	(Minowa et al. 1998)
	Bagasse	49.20	74.98	19.54	(Minowa et al. 1998)
	Bagasse pith	45.18	72.16	22.13	(Minowa et al. 1998)
	Corn stalk	42.43	68.18	21.73	(Minowa et al. 1998)
	Coconut husk	30.55	56.45	38.82	(Minowa et al. 1998)
	Almond shell	50.7	28.9	20.4	(Demirbas 2004)
	Coconut shell	26.49	79.29	35.54	(Minowa et al. 1998)
	Wood bark	24.8	29.8	43.8	(Demirbaş 1997)
	Beech wood	45.3	31.2	21.9	(Demirbas 2004)
	Hazelnut seed coat	29.6	15.7	53	(Demirbaş 1997)
	White poplar	49.0	25.6	23.1	(Yu et al. 2008)
	Coconut Shell	22	26	46	(Yang et al. 2006)
	Fiber	19	37	33	(Yang et al. 2006)
	White willow	49.6	26.7	22.7	(Yu et al. 2008)
	Empty Fruit Bunch	26	43	24	(Yang et al. 2006)
	Corncob	50.5	31	15	(Demirbas 2004)
	Wheat straw	28.8	39.4	18.6	(Demirbas 2004)
	Monterey pine	41.7	20.5	25.9	(Yu et al. 2008)
	Walnut shell	25.6	22.7	52.3	(Demirbas 2004)
	Douglas fir	42.0	23.5	27.8	(Yu et al. 2008)
	Tea waste	30.2	19.9	40	(Demirbas 2004)
	Hazelnut shell	26.8	30.4	42.9	(Demirbas 2004)
	Sunflower shell	48.4	34.6	17	(Demirbas 2004)
	Olive husk	24	23.6	48.4	(Demirbas 2004)
	Spruce wood	49.8	20.7	27	(Demirbas 2004)
Plant species	European birch	48.5	25.1	19.4	(Yu et al. 2008)
	Kenaf	42.60	81.27	10.31	(Minowa et al. 1998)
	Acacia Mangium	43.12	72.14	29.91	(Minowa et al. 1998)
	Coastal Bermuda grass	25	35.7	6.4	(Sun and Cheng 2002)
	chinquapin	46	20	30	(Ando et al. 2000)
	Japan cedar	35	24	33	(Ando et al. 2000)
	Switch grass	45	31.4	12	(Sun and Cheng 2002)
Environmental wastes	Newspaper	40–55	25–40	18–30	(Yu et al. 2008)
	Waste material	50.6	29.2	24.7	(Demirbaş 1997)

Understanding the proximate and elemental composition of lignocellulosic biomass is essential for optimizing CNC production, as these characteristics directly influence the efficiency and quality of the final product.

## Process overview for CNC production from lignocellulosic biomass

The composition of lignocellulosic biomass plays a critical role in the formation of cellulose nanocrystals (CNCs). Biomass rich in cellulose and hemicellulose tends to produce higher CNC yields, while a higher lignin content can reduce CNC yields (Johar et al. 2012). Understanding the structure of cellulose fibers within lignocellulosic biomass is essential for optimizing CNC production (Haldar & Purkait 2020). Table 2 provides an overview of the lignocellulosic composition of various biomass sources, helping to identify the most suitable feedstocks for CNC synthesis. In general, biomass sources with high cellulose and low lignin content yield the greatest amounts of CNC.

**Fig. 1:**
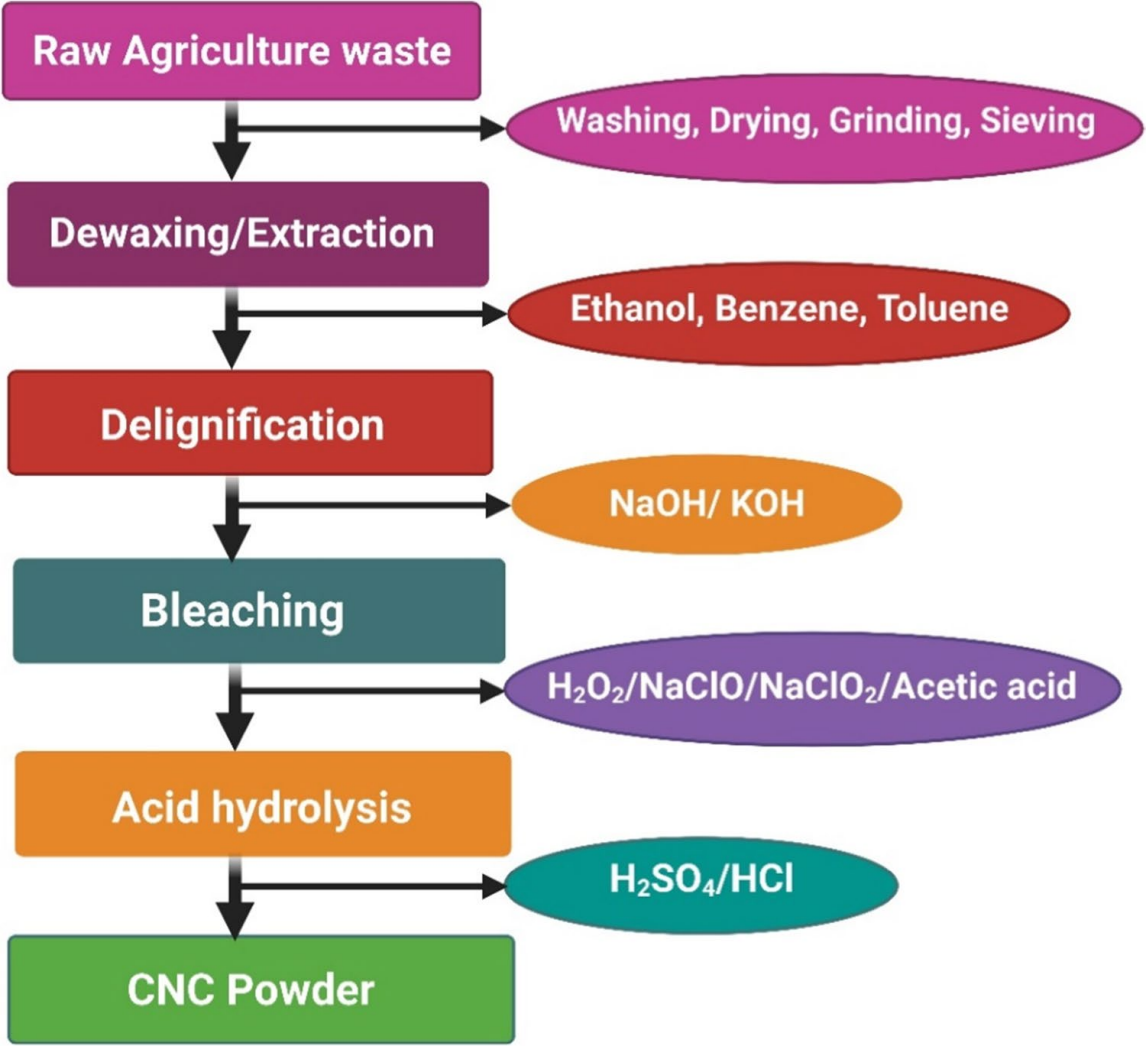
Steps involved in the conventional method of CNC synthesis from agricultural wastes

Figure 1 outlines the key steps involved in CNC production. The process begins with the washing and drying of raw biomass to remove surface impurities and soluble contaminants. The biomass is then ground and screened to ensure uniform particle size. Following this, the material undergoes alkali treatment to remove lignin (delignification) and hemicellulose, reducing cellulose polymerization. Purification is achieved through bleaching, where agents such as sodium chlorite (NaClO₂), sodium hypochlorite (NaClO), hydrogen peroxide (H₂O₂), and acetic acid are used. CNCs are then extracted through depolymerization using acids such as sulfuric acid (H₂SO₄), hydrochloric acid (HCl), phosphoric acid (H₃PO₄), or hydrobromic acid (HBr) (Alvira et al. 2010). After hydrolysis, the CNCs are washed, centrifuged, and dialyzed to remove residual acids. The CNC suspension is stabilized by sonication and finally dried to obtain a powdered form. These steps are discussed in detail in the following sections.

### Biomass pretreatment

Biomass pretreatment is a critical step in CNC production, as it removes non-cellulosic components and enhances the accessibility of cellulose for further processing (Phanthong et al. 2018). Effective pretreatment increases the efficiency of subsequent steps by reducing the recalcitrant nature of lignocellulosic biomass and improving cellulose extraction. Both physical and chemical methods are used, each contributing uniquely to the overall process.

#### Physical pretreatment methods

Physical pretreatment methods aim to reduce the size and uniformity of biomass particles, making them more manageable for chemical treatments. These methods include chipping, grinding, and milling, which break down the biomass into smaller particles (Lee et al. 2014). By achieving a consistent particle size, physical pretreatment ensures that the subsequent chemical processes, such as delignification and hydrolysis, are more efficient and uniform across the material.

#### Chemical pretreatment methods

Chemical pretreatments are crucial for breaking down the rigid structure of lignocellulosic biomass, making it easier to extract cellulose. This step involves the removal of non-cellulosic components, such as hemicellulose, lignin, and other impurities, which are typically more resistant to degradation. The key chemical treatments used in CNC production include dewaxing, delignification, and bleaching (Alemdar & Sain 2008, Johar et al. 2012; Li et al. 2009, Lu and Hsieh 2012).

##### Dewaxing

The first step in chemical pretreatment is dewaxing, which removes waxy substances such as phenolics, oils, waxes, and pigments that coat the biomass surface (Ghorbani et al. 2015). Removing these waxy layers is important for improving the efficiency of subsequent treatments by exposing the cellulosic fibers.

##### Delignification

Delignification is one of the most critical steps in CNC production, as it removes the lignin—a non-cellulosic, amorphous component that surrounds cellulose fibers. This process also removes hemicellulose, silica, and other impurities, ensuring higher cellulose purity. Delignification methods such as oxygen delignification (Ji et al. 2007) and alkaline hydrogen peroxide delignification (Valim et al. 2017) are commonly used. Oxidative delignification, which employs reagents such as ozone gas, dioxygen, sodium chlorite, chlorine dioxide, and chlorine, releases free radicals that efficiently break down the lignin structure, facilitating the removal of the lignocellulosic matrix.

##### Alkaline treatment

Among the various pretreatment methods, alkaline hydrogen peroxide treatment is particularly effective for a wide range of lignocellulosic biomasses. This treatment enhances the solubility of hemicellulose by cleaving bonds through saponification reactions (Pedersen & Meyer 2010). Alkaline delignification is pH-dependent, with optimal hydrogen peroxide decomposition typically occurring at a pH of 11.5—11.6 (Gould 1985; Palamae et al. 2014). This method not only removes lignin but also improves the overall efficiency of subsequent enzymatic hydrolysis.

#### Bleaching

After delignification, bleaching is employed to remove any residual hemicellulose and lignin from the biomass. Bleaching agents such as sodium chlorite (NaClO₂) or sodium hypochlorite (NaOCl) are used to break down phenolic molecules containing chromophoric groups in lignin, which can give biomass a darker colour (Dufresne 2017). The exact bleaching process depends on the cellulose source, with adjustments made based on the characteristics of the specific biomass being processed. The goal of bleaching is to increase the purity of the cellulose and ensure that the final CNC product is of high quality.

In summary, pretreatment plays a foundational role in the production of CNCs by ensuring that non-cellulosic components are removed, and the cellulose is ready for hydrolysis. Without these crucial steps, the efficiency of CNC extraction would be significantly reduced, affecting both yield and quality.

### Methods of CNC synthesis

The synthesis of CNC from lignocellulosic biomass can be achieved through various hydrolysis methods, each offering distinct advantages and challenges. This section provides an overview of these methods, focusing on the key steps and considerations for each approach.

#### Acid hydrolysis

Acid hydrolysis is a widely used technique for producing CNC by breaking down cellulose fibers (Espino et al. 2014). This process typically involves five key steps (Anwar et al. 2021a; Ilyas et al. 2018; Kian et al. 2018). First, the hydrolysis reaction is initiated in an acidic medium under controlled conditions, including the ratio of acid to cellulose, stirring speed, acid concentration, and temperature (Hafid et al. 2021). The reaction is then stopped by washing the fibers with water, followed by centrifugation to concentrate the cellulose (Villar et al. 2024). In the third step, dialysis with deionized water removes residual acids. Sonication is then employed to ensure even dispersion of CNC particles. Finally, the suspension is dried, yielding solid CNC (Di Giorgio et al. 2020). Table 3 provides an overview of CNC production protocols via inorganic acid hydrolysis, including yields and quality from various biomass sources.

**Table 3:** Cellulose nanocrystals (CNC) production protocols using inorganic acid hydrolysis

Material	Conditions	Findings	References
Microcrystalline cellulose (MCC) of roselle Fiber	Acid hydrolysis was carried out using solid: Liquid of 1:32 with 50% (w/w) of H_2_SO4 at 82 ◦C for 45 min	• Significant cellulose the crystallinity of 79.5% was observed • Significant removal of hemicellulose and lignin	(Kian et al. 2018)
Wood sawdust	Acid hydrolysis was conducted with 60% (w/ w) H_2_SO_4_ at solid: liquid of 1:10. The temperature was fixed at 60 °C for 60 min of the reaction period	• Higher reaction rate • Increase biomass accessibility • Applicable for several types of biomass	(Shaheen and Emam 2018)
Microcrystalline cellulose (MCC) of cotton linter	2 g of MCC was introduced into the reaction vessel containing 1 M of amberlite IR120 in 250 ml of water and the reaction was continued for 1 h at 80 ◦C under rigorous stirring	Single step process resulted into the production of spherical type CNC • Final product does not necessitate of any downstream processing like dialysis or sonication • Higher thermal stability • Yield of 94% reported for CNC products	(Ahmed-Haras et al. 2020)
Rice husk	Following delignification and bleaching, 200 g of wet biomass was treated with 4 mol/L H_2_SO_4_ at 60 ^o^C for 60 min under continuous stirring	• Substantial CNC yield of 95% was reported • Significant cellulose recovery of 90% was observed	(Islam et al. 2018)
Brown sea weed	Acid hydrolysis was conducted with 51% of H_2_SO_4_ using solid: liquid of 1:15 at 45 °C for 30 min	• Conventional processes facilitate to produce NCCs with higher crystallinity (66.9%–98.8%) • Thermally stable CNCs exhibit every potential to use as a filler in packaging system	(Doh et al. 2020)

#### Mineral acid hydrolysis

Mineral acid hydrolysis is frequently used for CNC production due to its ability to break the 1,4 β-glycosidic bonds in cellulose, resulting in compact crystalline structures that resist further degradation (Xue et al. 2021). While the crystalline regions remain largely intact, the amorphous regions are partially depolymerized, facilitating the production of CNC (Haldar et al. 2016). Hydrosulfuric acid (H₂SO₄), hydrobromic acid (HBr), hydrochloric acid (HCl), and phosphoric acid (H₃PO₄) are the most commonly used mineral acids for CNC synthesis (Raza and Abu-Jdayil 2022, Teo & Wahab 2020). Of these, H₂SO₄ is particularly favored due to its ability to produce stable CNC suspensions with negatively charged sulfate groups that aid in CNC dispersion (Listyanda et al. 2020). Table 4 provides an overview of CNC synthesis using H_2_SO_4_.

**Table 4:** Mineral-acid-hydrolyzed CNC production processes

Lignocellulosic biomass	Delignification Process	Bleaching process	Acid hydrolysis	Findings	References
Sugarcane bagasse	Steam explosion: 195 °C, 15 min	Repeated 7 times with 1.4% (w/v) sodium chlorite	60% (v/v) H_2_SO_4_, solid: liquid = 1:20, 45 °C, 75 min, 700 RPM	Cellulose content before final hydrolysis 87.68 wt.%, crystallinity index = 68.28% and length: width = 200:20 nm/nm)	(Sukyai et al. 2018)
Oil palm empty fruit bunch	4% NaOH, 80 ˚C, 2 h	150 mL dimethylsulfoxide, 80 °C	64% (w/v) H_2_SO_4_, 45 C, ultrasound bath, 2 h sonication	Cellulose content > 90 wt. %, crystallinity index = 80%, and spherical CNC with average diameter = 30–40 nm	(Azrina et al. 2017)
Rice husk	4 M NaOH, 80 °C, 24 h, 400 RPM	15 wt.% NaClO, 60 °C, 60 min, 800 RPM	4 M H_2_SO_4_, 60 min, 60 °C, 800 RPM	Cellulose content before final hydrolysis = 97 wt.%, crystallinity index = 61.6% and length: width = 550:50 nm/nm)	(Islam et al. 2017)
Date palm waste	10 wt/wt NaOH, 160 °C, 2 h	-	20 v/v% H_2_SO_4_, 120 C, 2 h	42–82 nm spherical particle, 11–19 m V (zeta potential)	(Mehanny et al. 2021)
Pine wood and corncob	250 mL solution of CH_3_COOH (93 wt.%), HCL (0.3 wt.%) and DI H_2_O (6.8 wt.%), 115 °C, 3 h	4% (w/v) NaOH, 24% (v/v) H_2_O_2_, 150 min for pine wood and 210 min for corncob, 50 °C	62 wt.% H_2_SO_4_, fiber: acid = 1:10, 44 °C, 90 min, 600–650 RPM	Crystallinity index = 78.2% for pine wood and 73.9% for corncob, spherical CNC with average diameter = 220 nm for pinewood and 43.8 nm for corncob	(Ditzel et al. 2017)
Corncob	2 wt. % NaOH, 100 °C, 4 h	27 g NaOH, 75 mL glacial acetic acid, 1.7 wt. % NaClO_3_	15 mL of 9.17 M H_2_SO_4_, 45 C, 30–90 min	Cellulose content before final hydrolysis > 66 wt. %, crystallinity index = 83.7% and (length: width = 210:4.15)	(Silvério et al. 2013)
Rice Straw	1.4% acidified NaClO_2_, pH 3–4 by CH_3_COOH, 70 °C, 5 h	5% KOH, 2 h, 90 °C	64–65 wt.% H_2_SO_4_, 8.75 mL/g acid to cellulose ratio	Crystallinity index = 91.2% and CNC (length: width = 117:11.2 nm/nm)	(Lu and Hsieh 2012)
Date palm trunk	Supercritical CO_2_ extraction, 340 bar, 90 °C, 20 wt/vol% NaoH, 90 °C, 6 h	NaClO_2_ with pH = 3.5, 70 °C, 4 h	Twin-screw extruder followed by sulphuric acid hydrolysis (50 wt. %)	20–60 nm irregular shape, 71.8% crystallinity index, high thermal stability = 280–290 °C	(Shaikh et al. 2021)
Rice husk	4 mol/L NaOH (2000 ml), 80 °C, 12 h, 400 RPM	500 ml NaClO (15%), 60 °C, 800 RPM, 60 min	4 mol/L H_2_SO_4_, 60 min, 60 °C, 800 RPM	Cellulose content before final hydrolysis = 95 wt.%, crystallinity index = 65% and (length: width = 275:35)	(Islam et al. 2018)
Date palm fibers	8 wt% NaOH, 25 °C, 25 min	10 w/v% NaClO_2_, 70–80 °C, 1 h	Sulphuric acid/ acetic acid in different ratios (10:90, 20:80, 30:70), 40–50 °C, 1 h	High crystallinity = 84.2%, high thermal stability = 230–290 °C	(Alothman et al. 2021)
Sugar palm fibers	70 °C, 28 mL acetic acid, 56 g NaCl, 7 h	500 mL 5% (w/v) NaOH, 2 h, 25 °C	60 wt.% H_2_SO_4_, 1200 RPM, 45 min, 45 °C, cellulose:H_2_SO_4_ = 1:20, sonication = 30 min	Cellulose content before final hydrolysis > 83 wt.%, crystallinity index = 85.9% and (length: width = 130:09 nm/nm)	(Ilyas et al. 2018)
Olive fiber	5% (w/v) NaOH, fiber/NaOH = 1:50, 1 h, 24 °C	2% (w/v) NaClO_2_, 1 h, 80 °C, fiber/sol = 1:50, pH = 4 with CH_3_COOH	35 wt.% H_2_SO_4_, 40–50 °C, fiber/acid ratio = 1:20	Cellulose content = 86.2 wt.%, crystallinity index = 83.1% and (length: width = 124.16:6.92) nm	(Kian et al. 2020)
Pineapple crown waste	5% NaOH, 90 °C, 1 h	16% (v/v) H_2_O_2_, 5% NaOH, 55 °C, 90 min	60 wt.% H_2_SO_4_, 45 °C, 1 h	Crystallinity index = 73% and CNC (length: width = 245:39 nm/nm)	(Prado and Spinacé 2019)
Pistachio shells	2 (w/v) NaOH, 100 °C, 2 h, repeated four times	1.7% NaClO_2_, buffer solution (2.7 g NaOH and 7.5 mL glacial acetic acid), 80 °C, 6 h	64 wt.% H_2_SO_4_, 45–50 °C, 90 min stirring	Cellulose content > 79.4 wt.%, crystallinity index = 83.1%, average spherical diameter 68.8 nm	(Kasiri and Fathi 2018)
Oil palm fronds	1% (w/v) MgSO4, 2.5% NaOH (w/v), 0.02% (w/v) AQ, 1.5% (w/v) H_2_O_2_, 90 °C, 30 min	1% (w/v) MgSO4, 2% NaOH (w/v), 0.02% (w/v) AQ, 1.2% (w/v) H_2_O_2_, 95 °C	8.75 mL of H_2_SO_4_ per gram of cellulose, 15 min	Cellulose content > 96.50 wt.%, crystallinity index = 78.5% and (length: width = 106:8 nm/nm)	(Dungani et al. 2017)
Sugarcane bagasse (SCB) and pinewood sawdust (PWS)	2 wt.% NaClO_2_ (80% pure), CH_3_COOH (99.8% pure), 65 °C, 8 h	5–10 wt.% NaOH, 3–5 h	45 wt.% H_2_SO_4_ (98% purity), 60–90 °C, 60 min	Crystallinity index = 93.77% for SCB and 86.24% for PWS, spherical CNC with average diameter = 3–10 nm for SCB and 20–60 nm for PWS	(Macías-Almazán et al. 2020)
Walnut shell	2 wt.% NaOH, biomass/ NaOH = 10 g/100 mL, 100 °C, 4 h	1.7 wt.% NaClO_2_, biomass/NaClO_2_ = 5 g/100 mL, 80 °C, 6 h	64 wt.% H_2_SO_4_, 45 °C, 1 h, vigorous stirring	Cellulose content > 87.9 wt.%, crystallinity index = 40.1% and (length: width = 55.49 nm/nm)	(Zheng et al. 2019)

Hydrochloric acid (HCl) is also effective in producing CNC with enhanced thermal stability, although the resulting suspensions are unstable and prone to flocculation (Xie et al. 2018). Phosphoric acid, a milder acid, can also be used for CNC production (Hastuti et al. 2018). Wang et al. (2019b) proposed a method using phosphoric acid to hydrolyze Pueraria root residue, achieving a CNC yield at 80 wt.% phosphoric acid. The resulting CNC exhibited good thermal stability and was well-dispersed in suspension, attributed to a high zeta potential of − 25.6 mV. The crystallinity index was 64%, with a length-to-diameter (L/D) ratio of 55. Hydrobromic acid is another strong acid used for CNC production (Wang et al. 2019b). In a study by Sucaldito and Camacho (2017), CNC was synthesized from freshwater algae using HBr at a concentration of 2.5 M, under conditions of 80 °C for 3 h, followed by ultrasonic treatment. The resulting CNC exhibited a high crystallinity index of 94%, a thermal decomposition temperature of 381.6 °C, and an average particle diameter of 20 nm.

In summary, while mineral acid hydrolysis is highly effective for CNC production, its scalability is limited by challenges such as equipment corrosion, high water usage, and environmental concerns (Gan et al. 2017; Sánchez et al. 2016; Zhu et al. 2016).

#### Organic acid hydrolysis

To address the challenges associated with mineral acid hydrolysis, such as equipment corrosion and environmental concerns, organic acids have been explored as an alternative for CNC production (Jiang et al. 2021; Robles et al. 2020; Wang et al. 2019a). Organic acids like oxalic acid are less corrosive, more environmentally friendly, and easier to recycle. However, organic acid hydrolysis generally requires higher reaction temperatures and longer processing times due to their lower acidity (Xu et al. 2017).

In a comparative study, the use of diluted oxalic acid and sulfuric acid for CNC production was examined. The results indicated that oxalic acid hydrolysis achieved an 85% CNC yield, compared to only 35% with sulfuric acid, showing a significantly higher yield (Xu et al. 2017). Furthermore, CNC produced using oxalic acid demonstrated superior thermal stability, with a degradation temperature of 350 °C, compared to 200 °C for sulfuric acid. Table 5 provides a summary of CNC production protocols using organic acid hydrolysis.

**Table 5:** CNC production protocols using organic acid hydrolysis

Organic acid	Material	Conditions	Results	References
Oxalic acid/H_2_SO_4_ combined	Eucalyptus kraft pulp	Oxalic acid:H_2_SO_4_:H_2_O = 5:1:4, 80 °C, 2–5 h	Aspect ratio = 20, CNC yield > 70%, crystallinity index = 78.1%, thermal degradation temp. = 363 °C	(Xie et al. 2019)
Acetic acid	Cotton cellulose	Acetic acid:octonal-1 = 9:1 (v/v) in the presence of phosphotungstic acid, 117 °C, 60 min	Max CNC yield = 34 with respect to original cellulose, crystallinity index = 89%, T_max_ = 360 °C	(Torlopov et al. 2018)
FeCl_3_-catalyzed formic acid	Eucalyptus kraft pulp	FeCl_3_ (0.005–0.025 M), 90 mL formic acid, 95 °C, 6 h, 400 RPM	CNC yield = 74%, crystallinity index = 70%, average particle size = 74 nm	(Du et al. 2016)
Oxalic acid	Waste filter paper	5.75–11.75 g oxalic acid/g filter paper, 100 C, 300 RPM, reaction time = 30–120 min	Max. yield CNC = 93.74%, crystallinity index = 86.64%	(Jia and Liu 2019)
Formic acid	Corncob pulp	0.5 wt.% HCL in mixture, 88 wt.% formic acid, liquid: solid = 30 mL/g, 95 °C	CNC yield = 66.3%, aspect ratio = 70, crystallinity index = 64%, max. degradation temperature = 360 °C	(Liu et al. 2016)

While organic acid hydrolysis presents several advantages, such as lower environmental impact and better recyclability, the longer reaction times and higher temperatures required may limit its scalability.

#### Solid acid hydrolysis

Solid acid hydrolysis has emerged as a promising alternative to liquid acid methods, addressing some of the issues related to acid recyclability and equipment corrosion. Solid acids are less corrosive, and their recovery and reuse are more straightforward compared to liquid acids. Despite these advantages, studies on CNC production using solid acid hydrolysis are still limited.

Research indicates that CNC produced through solid acid hydrolysis tends to have higher yields and improved thermal stability(Raza and Abu-Jdayil 2022). However, one major limitation is the longer reaction times required compared to traditional mineral acid hydrolysis. This method holds potential for further exploration, particularly in terms of optimizing reaction conditions and improving the economic feasibility. Table 6 summarizes key findings from studies on CNC production using solid acid hydrolysis.

**Table 6:** CNC production via solid acid hydrolysis

Solid acid	Material	Conditions	Results	References
1 D lignin-based activated carbon fiber solid acid catalyst (HTSACFs)	Rice straw	Cellulose pulp: HTSACFs = 1:4, 110–150 °C, 24 h, pressure = 1.4–1.8	At 150 °C and 5 atm; Crl = 72.2%, 68.9% of cellulose got hydrolyzed (64% glucose and 8.1% nanocellulose), nanocellulose with L:W:T = 1:3.1:2.1 µm, more selectivity toward glucose, catalyst was recovered after hydrolysis	(Hu et al. 2015)
Phosphotungstic acid (H_3_PW_12_O_40_)	Bleached hardwood pulp	50–85 wt.% H_3_PW_12_O_40_, 90 °C, 15–30 h, 0.5 g wood pulp and 40 mL HPW	75 wt.% H_3_PW_12_O_40_ at 90 °C and 3 h; Crl = 85%, CNC width = 14–50 nm and length of several nanometers, thermal decomposition at 350 °C, H_3_PW_12_O_40_ was recovered	(Liu et al. 2014)
Phosphotungstic acid (PTA)/acetic acid	Cotton cellulose	PTA (1.2 g m/L) mix. With H_2_O_2_, PTA sol. mix. with MCC in acetic medium, 3 h, 110 °C	Rod-like CNCs were obtained, L:D = 300:10, aspect ratio = 30, thermal degradation temperature = 275 °C, Crl = 91.9%	(Torlopov et al. 2017)
Cation exchange resin (NKC-9)	Microcrystalline cellulose	MCC:NKC-9 = 1:10, reaction in ultrasonication, 48 °C, 189 min, 300 RPM	CNC yield = 50.04%, CNC L:D = 100:10, aspect ratio = 10, Crl = 84.265 for NKC-9 CNC and 77.29% for H2SO4 CNC, resin catalyst was easily separated from the CNC suspension	(Tang et al. 2011)

#### Enzymatic hydrolysis

Enzymatic hydrolysis has garnered interest as a more environmentally friendly and sustainable approach to CNC production. Unlike acid-based methods, enzymatic hydrolysis avoids the production of toxic waste and consumes less energy, making it a greener alternative (Zhang & Lynd 2004). Additionally, enzymatic processes operate under milder conditions, reducing the need for high temperatures and pressures.

Enzymatic hydrolysis involves the use of cellulases, which selectively cleave the β-1,4-glycosidic bonds in cellulose, producing CNC with excellent structural integrity. This method also minimizes the formation of unwanted byproducts, such as sugars, and reduces the wastage of chemicals (Raza and Abu-Jdayil 2022). Table 7 presents a summary of studies focused on enzymatic hydrolysis for CNC synthesis, highlighting the superior physicochemical properties and thermal stability of CNC produced using this method.

**Table 7:** Overview of enzymatically hydrolyzed CNC production

Enzymatic medium	Feedstock	Conditions	Results	References
Cellulose viz Clast (mostly endoglucanase)	Bamboo fibers	2 wt.% pulp concentration mixed with 0.1 wt.% cellulose, 2 h, 50 °C, stirred after every 20 min	Nanocellulose having L:D = 221:5.3, aspect ratio = 42, cheaper than conventional methods (compared to H_2_SO_4_ in this study)	(Zhang et al. 2012)
Cellulase viz. cerrena sp. Fungus	Cotton inters	Cellulase dose = 20 U/g, treatment time = 2 h, 50 °C, pH = 5 maintained by 50 mM sodium acetate buffer solution	CNC yield = 80%, sulfur content < 1%, size = 200 nm, zeta potential = -50 mV, degree of polymerization = 200, profitable oligosaccharides can be utilized without harsh H_2_SO_4_ hydrolysis	(Raza and Abu-Jdayil 2022)
Cellic CTec2 (endoglucanase activity of 2625 U/gram)	Sugarcane bagasse	Solid loading = 15–20 wt.%, enzyme loading = 7.5–15 mg protein/g pulp, reaction time = 72–96 h	Crl = 90.3%, cellulose content = 91.8%, average particle size = 14–22 nm, thermal degradation temperature = 294 °C	(Pereira and Arantes 2020)
Cellulase (700 EGU/g); endoglucanase unit	Wheat straw	6 g microcrystalline cellulose mixed with 3 mL cellulase and 200 mL acetate buffer solution (pH = 4.8), 50 °C, 72–120 h	CNC yield = 22.57% at 120 h combined with ultrasonication, CNC L:W = 200:10, Crl = 87.46%, thermal decomposition temperature = 250 ˚C compared to 200 ˚C for H_2_SO_4_ hydrolyzed CNC in this study	(Cui et al. 2016)

Despite its advantages, enzymatic hydrolysis is still less efficient in terms of production time and yield compared to acid hydrolysis methods. However, with ongoing advancements in enzyme technology, this method may become more viable for large-scale CNC production.

#### Ionic liquid (IL) treatment

Ionic liquids (ILs) have gained attention as novel solvents for isolating nanocellulose from biomass materials. These compounds exhibit unique properties such as low vapor pressure, high chemical and thermal stability, non-flammability, and recyclability, making them ideal for sustainable CNC production (Grząbka-Zasadzińska et al. 2019; Lindman et al. 2010; Man et al. 2011; Tan et al. 2015). Furthermore, ionic liquids can dissolve lignocellulosic materials, facilitating the extraction of nanocellulose while avoiding the harsh conditions associated with traditional acid hydrolysis.

Many researchers have demonstrated the ability of ionic liquids to dissolve lignocellulosic materials, facilitating the extraction of nanocellulose (Alayoubi et al. 2020; Xie et al. 2012). One notable method involves the use of 1-ethyl-3-methylimidazolium acetate as an ionic liquid for delignification and defibrillation of wood pulp, achieving a CNC yield of 20 wt.% with a crystallinity index of 75% and an aspect ratio (L/D) of 65 (Abushammala et al. 2015). Table 8 provides an overview of recent research on CNC production using ionic liquid hydrolysis.

**Table 8:** Recent studies of CNC production using Ionic liquids

Raw material	Ionic liquid salt	Results	References
Blueberry residue	1-ethyl-3-methylimidazolium	Crl = 73% Avg. size = 46 nm	(Pacheco et al. 2020)
Cotton cellulose pulp	1-butyl-3-methylimidazolium chloride	Avg. size = 10–20 nm	(Wang et al. 2015)
Adansonia kilima (African baobab leaves)	1-butyl-3-methylimidazolium hydrogen sulfate	Crl = 86.46% Avg. size = 15–20 nm	(Chowdhury et al. 2019)
Sugarcane bagasse	1-butyl-3-methylimidazolium chloride	Crl = 69% Avg. size = 4–35 nm	(Sankhla et al. 2021)
Microcrystalline cellulose	1-butyl-3 methylimidazolium chloride	Crl = 73% Avg. size = 9 nm	(Iskak et al. 2017)
Miscanthus (silvergrass)	Triethylammonium hydrogen sulfate with 20% H2O as a cosolvent	Crl = 73% Avg. size = 3.8 nm	(Tu et al. 2020)
Cellulose powder	1-butyl-3-methylimidazolium chloride	Crl = 65.8% Avg. size = 10–25 nm	(Phanthong et al. 2017)
Calophyllum inophyllum (mastwood tree)	1-butyl-3-methylimidazolium chloride	Crl = 81% Thermal degradation = 200° C–350 °C	(Mawarni et al. 2022)

While ionic liquids offer several advantages, such as the ability to dissolve biomass under mild conditions and their low environmental impact, the high cost of ionic liquids and challenges in large-scale production remain barriers to widespread adoption.

## Characterization of CNC

Characterizing CNC is essential for evaluating its properties and determining its suitability for various industrial applications. A range of analytical techniques is employed to assess the thermal, structural, and chemical characteristics of CNC. Thermogravimetric analysis (TGA), derivative thermogravimetry (DTG), and differential scanning calorimetry (DSC) are commonly used to examine thermal stability and decomposition behavior by measuring weight loss under thermal conditions (Jordan et al. 2019). Additionally, atomic force microscopy (AFM), transmission electron microscopy (TEM), scanning electron microscopy (SEM), and field emission scanning electron microscopy (FESEM) are used to study the structural and morphological features of CNC, including its size and shape. Zeta potential measurements help determine surface charge and stability. X-ray diffraction (XRD) provides insight into the crystallographic structure, while Fourier transform infrared (FTIR) spectroscopy is employed to identify surface functional groups and analyze the chemical composition of CNC. The following sections explore the chemical, thermal, and structural properties of CNC in greater detail.

### Chemical properties

FTIR spectroscopy is one of the most widely used techniques for identifying and analyzing chemical groups in CNC samples, typically within the 400–4000 cm⁻^1^ range. The resulting spectra offer valuable insights into the molecular structure and chemical bonds present in the material. FTIR is particularly useful in CNC studies for comparing the chemical composition of raw biomass with that of the extracted CNC.

**Fig. 2:**
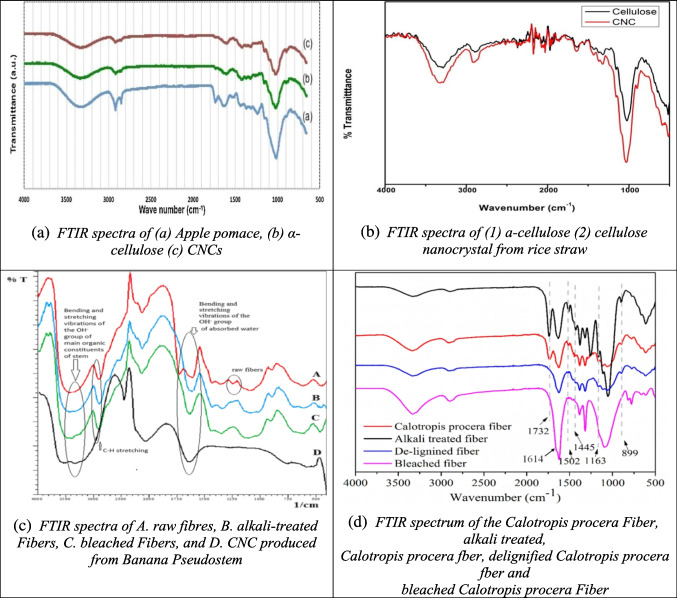

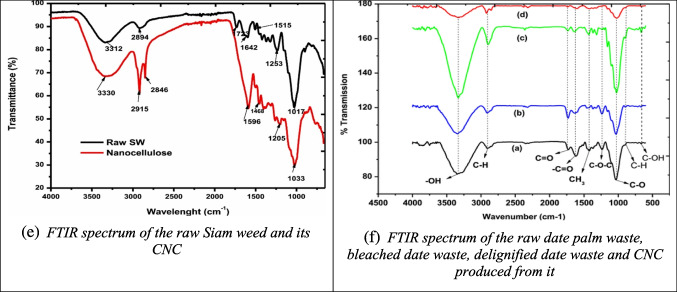
Some reported FTIR results of CNC from different biomass sources (**a**: apple pomace; **b**: rice straw; **c**: banana pseudostem; **d**: calotropis procera fiber; **e**: Siam weed; **f**: date palm waste) and their raw materials and intermediate products different studies (Melikoğlu et al. 2019; Ogunjobi et al. 2023; Raza et al. 2022; Song et al. 2019; Thakur et al. 2020; Zope et al. 2022)

For example, the FTIR spectrum of CNC derived from α-cellulose and apple pomace (Fig. 2a) shows significantly reduced peaks at wavenumbers 1748, 1530, and 1259 cm⁻^1^, which correspond to hemicellulose and lignin, indicating their effective removal during processing (Melikoğlu et al. 2019). In contrast, the CNC spectrum from rice straw (Fig. 2b) shows no new peaks after acid hydrolysis, with consistent bands at 3400, 2400, and 1060 cm⁻^1^, reflecting the presence of pure cellulose (Thakur et al. 2020).

Similarly, CNC from banana pseudostem and *Calotropis procera* fiber (Figs. 2c and d) exhibits a complete disappearance of peaks associated with hemicellulose and lignin (notably at 1730, 1250, and 1502 cm⁻^1^). Additionally, the reduction of bands in the 850–1500 cm⁻^1^ range, related to non-cellulosic crystalline structures, indicates the successful removal of non-cellulosic material (Song et al. 2019; Zope et al. 2022).

In the case of Siam weed (Fig. 2e), the CNC spectrum reveals an increased peak at 3330 cm⁻^1^, characteristic of CNC, while a peak at 1642 cm⁻^1^ corresponds to H–O–H bending vibrations from absorbed water, attributed to hydroxyl groups in cellulose (Ogunjobi et al. 2023). Finally, the FTIR spectrum of CNC from date palm waste (Fig. 2f) shows absorption bands between 800–900 cm⁻^1^, related to C–H and C–O stretching. The disappearance of the peak at 1728 cm⁻^1^, which represents C═O stretching from hemicellulose, confirms the successful removal of hemicellulose during treatment (Raza et al. 2022).

Overall, FTIR analysis across various biomass sources confirms the successful removal of non-cellulosic components, such as hemicellulose and lignin, during CNC extraction. This indicates the purity of the CNC produced and its suitability for further industrial applications.

### Thermal properties

Thermogravimetric analysis (TGA) is a commonly used technique for studying the thermal degradation behavior of biomass feedstocks and their components—such as cellulose, hemicellulose, and lignin—before thermochemical conversion. TGA measures mass loss as a function of increasing temperature and/or time (Bach & Chen 2017). The TGA curves generated from this analysis can be used to identify distinct degradation stages for each material.

**Fig. 3:**
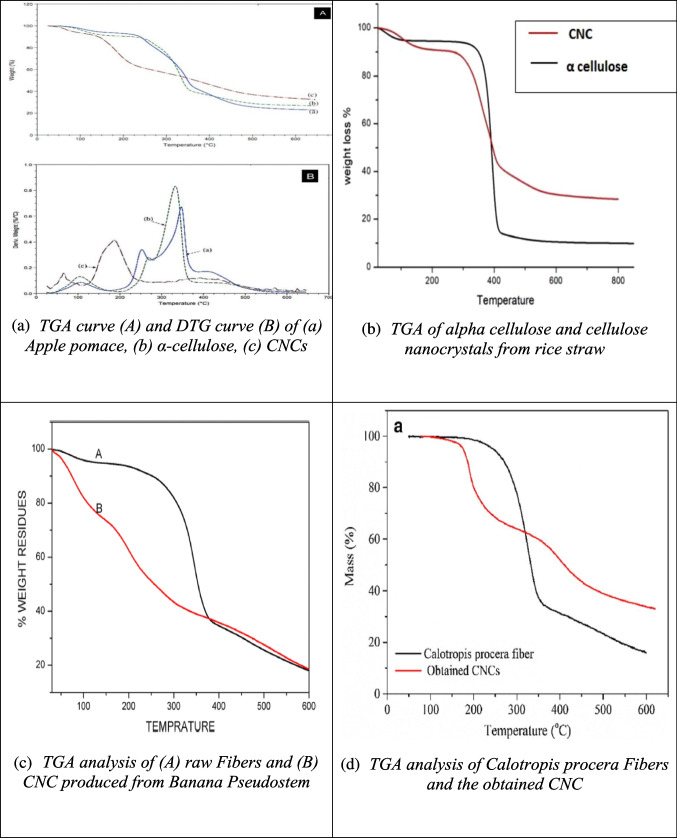

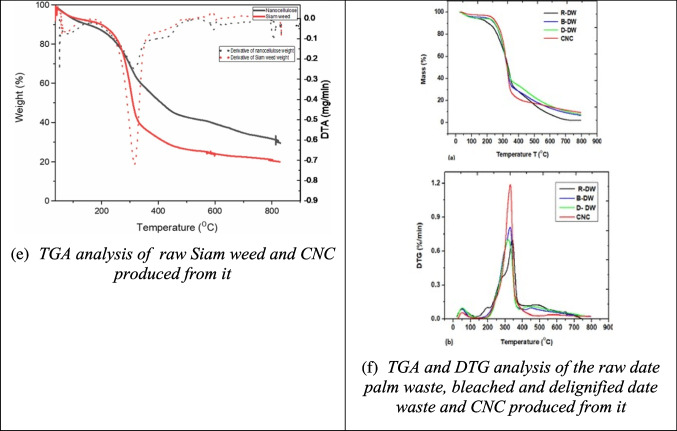
Some reported TGA and DTG results of CNC from different biomass sources (**a**: apple pomace; **b**: rice straw; **c**: banana pseudostem; **d**: calotropis procera fiber; **e**: Siam weed; **f**: date palm waste) and their raw materials and intermediate products different studies (Melikoğlu et al. 2019; Ogunjobi et al. 2023; Raza et al. 2022; Song et al. 2019; Thakur et al. 2020; Zope et al. 2022)

For instance, Fig. 3(a) shows that in all samples, the initial weight loss below 120 °C is due to moisture evaporation. In the case of apple pomace, approximately 45% of its weight is lost between 150–350 °C, while cellulose exhibits weight loss between 250–400 °C. CNC, however, shows its main weight loss between 150–300 °C, suggesting lower thermal stability compared to cellulose (Melikoğlu et al. 2019).

In contrast, Figs. 3(b), (c), and (d), which present the decomposition temperatures of CNC produced from rice straw, banana pseudostem, and *Calotropis procera* fiber, demonstrate higher thermal stability than cellulose. These CNC samples also display lower residual mass, indicating the successful removal of hemicellulose, lignin, and pectin, as well as higher cellulose crystallinity in the CNCs (Song et al. 2019; Thakur et al. 2020; Zope et al. 2022).

The TGA-DTG curve for raw Siam weed and its corresponding CNC, shown in Fig. 3(e), reveals three distinct degradation stages. The first stage, marked by maximum weight loss below 110 °C, corresponds to moisture evaporation. Degradation of the cellulose chain occurs between 200–350 °C, associated with the breakdown of hemicellulose and lignin (Ilyas et al. 2018). In the final stage, CNC degradation occurs at 450 °C, while the raw Siam weed degrades at 400 °C. The higher degradation temperature of CNC suggests greater kinetic stability compared to the raw biomass, contributing to its enhanced thermal stability (Ogunjobi et al. 2023). The lower thermal stability of raw Siam weed is attributed to the presence of non-cellulosic substances, which are removed during the CNC extraction process, leading to improved thermal stability in the final CNC product (Yang et al. 2007).

Lastly, Fig. 3(f) illustrates the thermal degradation behavior of CNC extracted from date palm waste, delignified, bleached and raw date palm waste. The raw material degrades in two stages, which are associated with the different percentages of lignin and hemicellulose present in the samples. After delignification, the thermal degradation temperature of date palm waste occurs at 227 °C, and bleaching increases the degradation temperature to 242 °C. The CNC shows improved thermal stability, with degradation occurring at 249.5 °C, which is attributed to the removal of hemicellulose and lignin during delignification and bleaching, as well as acid hydrolysis treatment (Wulandari et al. 2016).

In summary, TGA analysis across various biomass sources highlights that CNC generally exhibits improved thermal stability compared to raw materials, largely due to the removal of non-cellulosic components such as hemicellulose and lignin during extraction and treatment processes.

### Morphology and structural properties

**Fig. 4:**
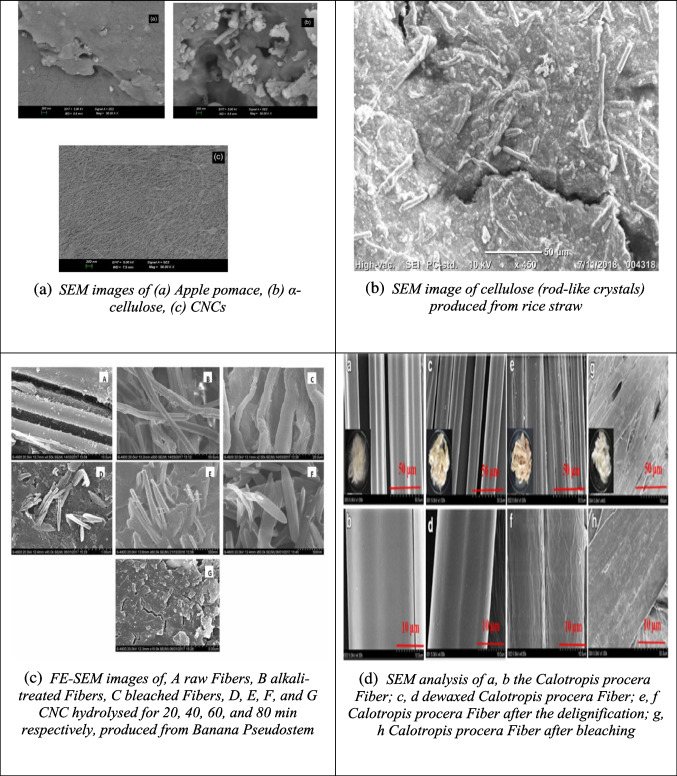
Some reported SEM results of CNC from different biomass sources (**a**: apple pomace; **b**: rice straw; **c**: banana pseudostem; **d**: calotropis procera fiber) and their raw materials and intermediate products different studies (Melikoğlu et al. 2019; Ogunjobi et al. 2023; Raza et al. 2022; Song et al. 2019; Thakur et al. 2020; Zhao et al. 2019; Zope et al. 2022)

Scanning electron microscopy (SEM) is a powerful technique used to analyze the structural characteristics of materials, such as size, shape, and texture, with magnifications up to 300,000x. In SEM, a focused electron beam scans across the sample, generating signals that are processed and displayed as high-resolution images. This technique is particularly well-suited for characterizing the nanoscale structure of CNC due to its excellent resolution. For example, Fig. 4(a) shows cellulose fibers from apple pomace, where the smooth surface suggests the effective removal of non-cellulosic materials (Melikoğlu et al. 2019). Acid hydrolysis of rice straw and banana pseudostem, as seen in Figs. 4(b) and (c), produces CNC in the form of tiny, rod-like crystals (Thakur et al. 2020; Zope et al. 2022). Similarly, Fig. 4(d) shows CNC extracted from *Calotropis procera* fiber, exhibiting a uniform needle-like shape with a length of 250 nm and a diameter of 12 nm (Song et al. 2019).

**Fig. 5:**
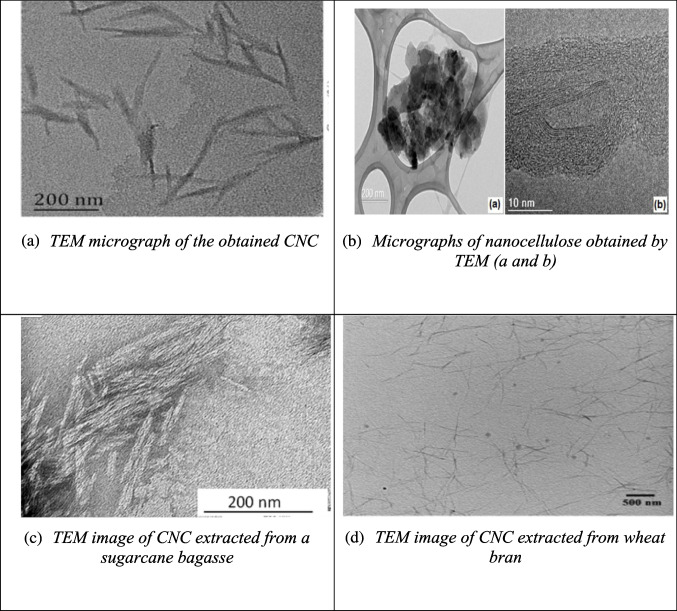

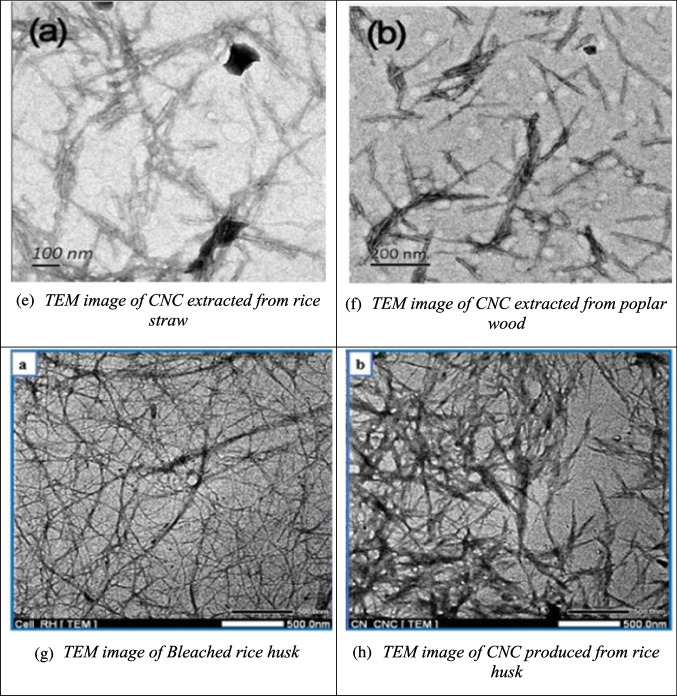
Some reported TEM results of CNC from different biomass sources (**a**: calotropis procera fiber; **b**: pineapple crown fibers; **c**: sugarcane bagasse; **d**: wheat bran; **e**: rice straw; **f**: poplar wood; **g**, **h**: rice husk) and their raw materials and intermediate products different studies (Faria et al. 2020; Khoo et al. 2018; Song et al. 2019; Vu et al. 2024; Xiao et al. 2019; Zhao et al. 2019)

While SEM provides surface-level details, transmission electron microscopy (TEM) allows for deeper visualization of CNC’s internal structure. TEM is widely used in nanomaterials research for its ability to provide high-resolution images by transmitting a focused electron beam through thin samples. This method is essential for analyzing the morphology of individual CNC particles. For example, Fig. 5(a) shows the TEM image of CNC from *Calotropis procera* fiber, displaying a uniform needle-like structure (12 nm in diameter and 250 nm in length) (Song et al. 2019). The CNC from pineapple crown fibers, shown in Fig. 5(b), reveals a distinct agglomerated, plate-like structure with a highly ordered interior surrounded by an amorphous carbon layer (Faria et al. 2020). CNC extracted from sugarcane bagasse and wheat bran are shown in Figs. 5(c) and (d), with diameters ranging from 80 to 100 nm (Khoo et al. 2018; Xiao et al. 2019). Figures 5(e) and (f) depict CNC from rice straw and poplar wood, which feature rod-like structures with slight aggregation. The CNCs from rice straw measure 9.1 nm in diameter and 145.8 nm in length, while the CNCs from poplar wood are 11.4 nm in diameter and 153.2 nm in length (Zhao et al. 2019). Figures 5(g) and (h) display the TEM images of bleached rice husk and CNC derived from rice husk. The bleached rice husk shows long fibrils with diameters between 1 and 10 nm, while the CNC has a rod-like structure formed after the removal of amorphous regions during acid hydrolysis, which also reduced the length of the fibrils (Vu et al. 2024).

**Fig. 6:**
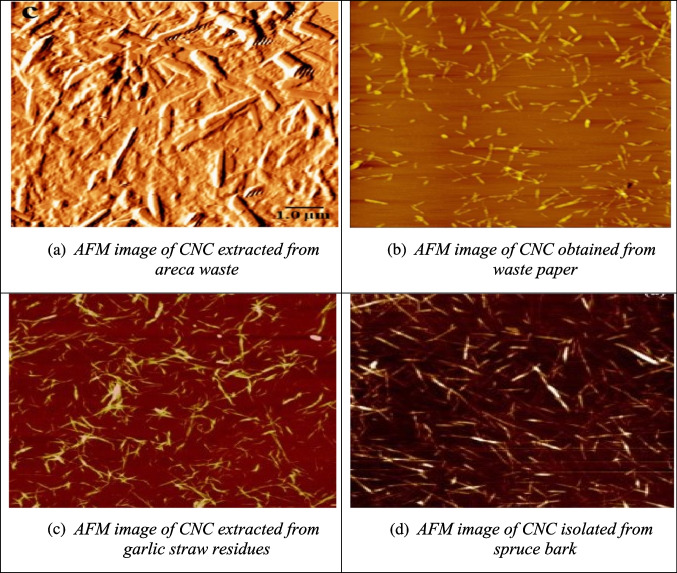
some reported AFM results of CNC from different biomass sources (**a**: areca waste; **b**: waste paper; **c**: garlic straw residues; **d**: spruce bark) (Guan et al. 2021; Kallel et al. 2016; Le Normand et al. 2014; Perumal et al. 2022)

In addition to SEM and TEM, atomic force microscopy (AFM) provides three-dimensional surface imaging at the nanoscale. AFM operates by scanning the surface with a cantilever equipped with a fine tip. The tip moves across the material with nanometric precision, providing a detailed surface map (Sousa & Scuracchio 2014). For example, AFM analysis of CNC from areca waste, as shown in Fig. 6(a), reveals a needle-like shape (Perumal et al. 2022). Figure 6(b) illustrates CNC from waste paper, exhibiting a regular whisker shape with diameters ranging from 20 to 40 nm and lengths of 400 to 800 nm (Guan et al. 2021). AFM images of CNC from garlic straw residues, presented in Fig. 6(c), show a well-defined rod-like shape with a diameter of 6 nm and a length of 480 nm (Kallel et al. 2016). Figure 6(d) depicts CNC from spruce bark, showing a rod-like structure with noticeable agglomeration, likely due to the high surface area and strong hydrogen bonding between the crystallites (Le Normand et al. 2014).

**Fig. 7:**
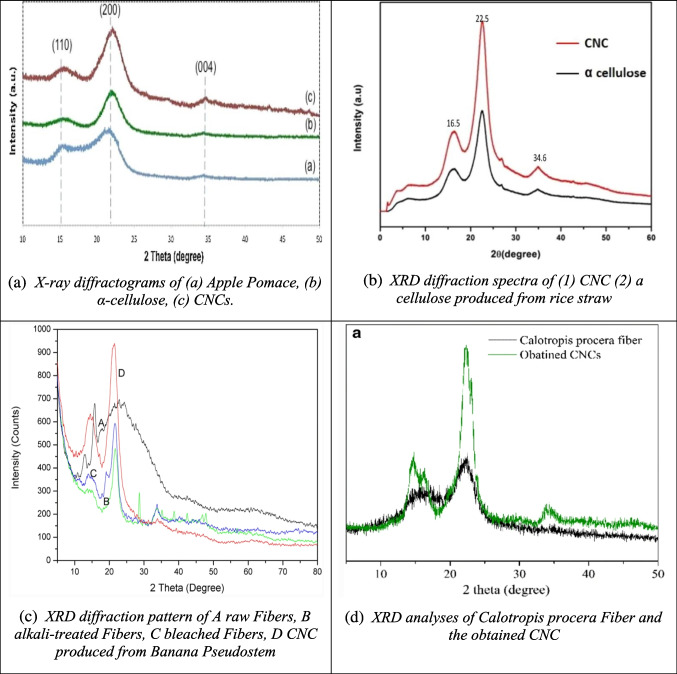
Some reported XRD results of CNC from different biomass sources (**a**: apple pomace; **b**: rice straw; **c**: banana pseudostem; **d**: calotropis procera fiber) and their raw materials and intermediate products different studies (Melikoğlu et al. 2019; Song et al. 2019; Thakur et al. 2020; Zope et al. 2022)

X-ray diffraction (XRD) is a key technique for evaluating the crystalline properties of CNC. XRD works by directing monochromatic X-rays at a sample, and the interaction between the X-rays and the crystalline structure produces diffracted rays. These rays are then measured at different angles to generate a diffraction pattern, which reveals details about the crystalline structure. Cellulose consists of both crystalline and amorphous regions, with the crystalline regions featuring tightly bound cellulose chains due to hydrogen bonding, while the amorphous regions lack this bonding. Figures 7(a) and (b) show the XRD analysis of apple pomace and rice straw, along with CNC produced from these biomasses. The presence of distinct crystalline peaks in all samples confirms the cellulose structure (Melikoğlu et al. 2019; Thakur et al. 2020). The crystallinity increased from 67 to 78% following the removal of hemicellulose and lignin from the amorphous regions of apple pomace and rice straw. Figure 7(c) shows a sharp crystalline peak in CNC produced from banana pseudostem, indicating the concentration of the crystal lattice (Zope et al. 2022). Similarly, Fig. 7(d) shows a significant increase in the crystallinity index of CNC from *Calotropis procera* fiber, suggesting that the amorphous hemicellulose was effectively removed through acid treatment (Song et al. 2019).

### Particle size

Zeta potential is a critical parameter for measuring the surface charge of nanomaterials. It reflects the electrostatic potential difference between the bulk solution and the nanoparticle surface. This technique involves passing a laser through a nanoparticle solution under an electric field, and the shift in the scattered light’s frequency—known as the Doppler Effect—is measured. Zeta potential is commonly used to assess changes in surface charge and evaluate the stability of nanoparticles during formulation (Parupudi et al. 2022).

**Fig. 8:**
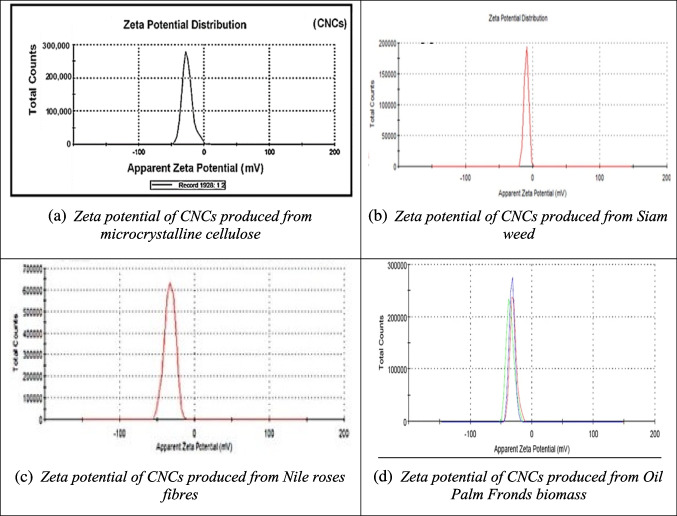
Some reported Zeta potential results of CNC from different biomass sources (**a**: microcrystalline cellulose; **b**: Siam weed; **c**: Nile Roses fibers; **d**: oil palm fronds biomass) (Dungani et al. 2017; Hemida et al. 2023; Ogunjobi et al. 2023; Whba et al. 2023)

For nanocellulose, a zeta potential value is considered stable if it is less than -30 mV or greater than 25 mV, as this prevents particle aggregation. In such cases, the electrostatic repulsion between nanoparticles in a colloidal solution leads to a more uniform particle distribution (Morais et al. 2013). Figures 8(a) and (b) illustrate the zeta potential of CNC produced from microcrystalline cellulose and Siam weed, both of which have negatively charged surfaces and a zeta potential of -9.57 mV. This value indicates a uniform suspension, achieved through strong particle repulsion (Ogunjobi et al. 2023; Whba et al. 2023). In addition, Fig. 8(c) shows CNC extracted from Nile roses fibers, which also displays a negative zeta potential. As the duration of acid hydrolysis increases, the zeta potential value decreases from -32.9 mV to -41 mV (Hemida et al. 2023). This negative zeta potential is linked to sulfate groups formed on the CNC chains during the acid hydrolysis process (Whba et al. 2023).

At shorter acid hydrolysis times, higher zeta potential values are observed, which enhance electrostatic repulsion and prevent CNC particles from aggregating (Yu et al. 2021b). Lastly, Fig. 8(d) presents CNC extracted from oil palm fronds biomass, with a high zeta potential of -32.73 mV, confirming the stability of the CNC suspension (Dungani et al. 2017).

Overall, the results demonstrate that CNC extracted from various biomass sources exhibits stable zeta potential values, which ensure particle stability and prevent aggregation in suspension.

## Applications of CNC

**Fig. 9:**
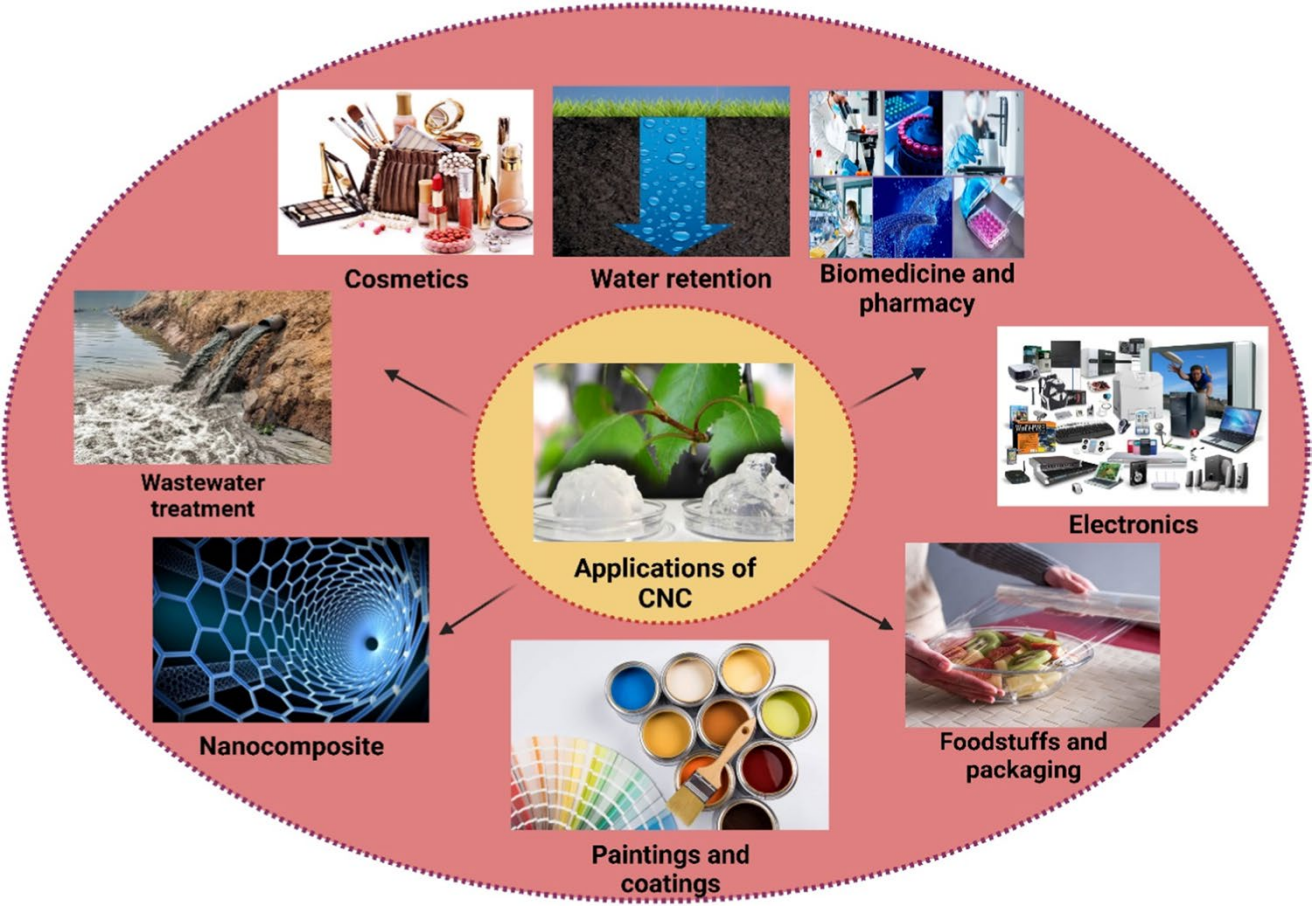
A variety of CNC applications

Researchers have utilized a variety of agricultural residues as raw materials for extracting environmentally friendly, high-value, and cost-effective cellulose nanocrystals (CNCs). Producing CNC from agricultural and forestry residues provides several environmental, social, and economic benefits. CNC has gained considerable attention due to its exceptional properties, including nontoxicity, high surface area, renewability, biocompatibility, excellent mechanical strength, low thermal expansion, strong water absorption, and superior retention capabilities (Ahmed et al. 2022; Mateo et al. 2021; Panaitescu et al. 2013). Additionally, chemical or physical surface functionalization of CNC allows for the incorporation of various functional groups, endowing CNC surfaces with distinct properties. This versatility broadens the potential applications of CNC across numerous fields, including food, pharmaceuticals, biomedicine, composites, optoelectronics, packaging, and cosmetics, among others. Among the many applications of CNC, wastewater treatment has emerged as one of the most promising. The following sections explore this and other key applications of CNC, as illustrated in Fig. 9.

### Wastewater treatment

Human activities significantly contribute to freshwater contamination through the disposal of biological waste, which negatively impacts marine ecosystems, microorganisms, and various organic and inorganic compounds. These pollutants not only degrade water quality but also disrupt marine habitats, causing long-term environmental damage. To mitigate these effects, CNC-based membranes and filtration methods have been developed for wastewater treatment (Panaitescu et al. 2013; Yu et al. 2021a).

CNC has proven to be highly effective in wastewater treatment due to its unique combination of properties, including high mechanical strength, water stability, large surface area, high aspect ratio, and surface charge. Specifically, CNC has been widely used in biogenic treatment processes, particularly for adsorbing harmful heavy metal ions such as Hg^2^⁺, U⁶⁺, Ag⁺, Ni^2^⁺, Cd^2^⁺, Pd^2^⁺, and Cu^2^⁺ from contaminated water (Albernaz et al. 2016; Kara et al. 2021; Sadare et al. 2022). The efficiency of CNC in adsorbing metal ions is attributed to its high hydroxyl (-OH) content, large surface area, and the presence of modified carboxyl (-COO⁻) groups, which provide specific interaction sites for binding metal ions. A higher number of negatively charged functional groups enhances the adsorption rate for these harmful metal ions. Moreover, CNC-based products have demonstrated significant potential in treating wastewater contaminated with harmful organic substances, including dyes (Chaka 2022), pharmaceuticals (Shahnaz et al. 2021), fertilizers (Potenza et al. 2022), and microorganisms (Perumal et al. 2022). Overall, CNC’s ability to remove both heavy metals and organic contaminants from wastewater makes it an excellent candidate for sustainable water treatment solutions.

### Foodstuffs and packaging

Conventional food packaging materials, such as polymers combined with polyethylene, present significant environmental challenges. These materials are not biodegradable and persist in the environment for extended periods. To address this issue, replacing harmful packaging materials with renewable, biodegradable alternatives can enhance environmental safety and reduce the risk of food contamination, especially when interacting with antimicrobial nanoparticles at active sites (Perumal et al. 2022; Reshmy et al. 2021). CNC has been applied as a packaging material to monitor food freshness, such as in chicken, by detecting color changes (Lu et al. 2020). As microbial decomposition and spoilage progress, the color of CNC-based packaging shifts from green to red, indicating that the food is no longer safe for consumption. This technology enables rapid, eco-friendly evaluation of food freshness (Seo et al. 2018).

Beyond monitoring freshness, CNC-based materials offer multiple benefits in food packaging. These include protecting food, reducing spoilage risks, extending shelf life (Mu et al. 2019), improving tensile strength, detecting harmful substances, and decreasing moisture and oxygen permeability in packaging films (Xiang et al. 2020). For example, the Young’s modulus (a measure of stiffness) of CNC/PA6 films increased by 250%, making them suitable for packaging and infiltration applications (Chaka 2022). Additionally, functionalized coated films designed to reduce harmful gas emissions during packaging provide properties such as thermal recyclability, bacterial resistance, and high transparency (Chaka 2022). These properties highlight the significant potential of CNC-based materials in creating sustainable, high-performance packaging solutions.

### Polymer nanocomposites

As biogenic nanomaterials, CNCs are ideal for forming polymer nanocomposites because of their exceptional mechanical properties, biodegradability, low density, sustainability, and nontoxicity. Integrating CNC into biopolymers accelerates the biodegradation of polymeric materials (Fortunati et al. 2015; Risite et al. 2022). For instance, de Souza et al. demonstrated that incorporating nanocellulose extracted from industrial and agricultural residues into polylactic acid (PLA) significantly enhances the tensile strength of biopolymer nanocomposites (de Souza et al. 2020). This improvement is due to CNC’s excellent dispersion and interaction with PLA’s functional groups, facilitating efficient stress transfer between fillers and matrix membranes.

Likewise, the incorporation of CNC into polyvinyl alcohol (PVA) has been shown to increase tensile strength by up to 140.2%, even with just 9 wt.% CNC, without compromising the homogeneity or transparency of the polymer matrix (Yadav et al. 2013). When CNC is combined with Chitosan/PVA, the transparency is preserved, and the tensile strength of the nano-bio-composite improves by 21% (Perumal et al. 2022). Moreover, this nano-bio-composite film shows strong antimicrobial activity against harmful postharvest fungi and foodborne bacteria. Another study found that adding 5 wt.% CNC to maleic anhydride-grafted polypropylene optimized tensile strength, modulus, and stiffness in the nanocomposite (Agarwal et al. 2020).

Several studies indicate that while light transmittance values decrease with the incorporation of CNC into polymer matrices, the mechanical properties, such as strength and stiffness, are significantly improved (Collazo-Bigliardi et al. 2018, Pal &Paul 2019). Finally, CNC-based polymer nanocomposites have been developed for a range of applications, including optoelectronics, catalysis, nanomedicine, water treatment, and environmental remediation (Barton & d’Errico 2012).

### Biomedicine and pharmacy

CNC’s exceptional properties—such as biodegradability, high surface area, inertness, ease of surface modification, strong mechanical strength, and biocompatibility—make it an ideal material for pharmaceutical and biomedical applications. CNC-based materials have been widely used in drug delivery systems for active molecules, including genes, plasmids, proteins, and drugs, as well as in tissue engineering (Chaka 2022). CNC’s high surface area-to-volume ratio, efficient cellular binding, biocompatibility, and low weight make it highly effective in drug delivery systems such as nanoparticles, cryogels, aerogels, membranes, and hydrogels (Mateo et al. 2021; Patel et al. 2020).

Engineered nanocellulose with bioactive and antimicrobial properties has been used for targeted drug delivery, especially in treatments for controlling bleeding (hemostasis) and wound healing (Patil et al. 2022). For instance, CNC materials incorporated into polycaprolactone (PCL)/chitosan/CNC nanocomposites have shown improved drug release capabilities for tetracycline. In contrast, pure PCL and chitosan exhibit limitations in drug release (Ruttala et al. 2017; Shrestha et al. 2021).

CNCs derived from softwood and functionalized with acetyl trimethylammonium bromide have been successfully used to deliver hydrophobic anticancer drugs such as etoposide, paclitaxel, and docetaxel, effectively killing KU-7 cancer cells over a 48-h period (Jackson et al. 2011). Additionally, rice husk-derived CNCs have shown promising antiproliferative and antioxidant properties, attributed to their interactions with phytochemicals like flavonoids, caffeic acid, p-coumaric acid, p-hydroxybenzoic acid, and ferulic acid (Gao et al. 2018).

The biocompatibility of CNCs has been further demonstrated in studies where human fibroblast skin cells immobilized on a polyvinyl alcohol (PVA)/CNC composite showed no harmful effects after 24 h of incubation, reinforcing CNC’s safety for biomedical applications (Lam et al. 2017; Xiong et al. 2017). These studies demonstrate the versatility of CNC in pharmaceutical applications, particularly in drug delivery and tissue engineering, highlighting its potential for further advancements in biomedicine.

### Cosmetics

In addition to its biomedical applications, CNC is extensively used in the skincare industry due to its stability, high viscosity, purity, biocompatibility, water-holding capacity, and shear-thinning properties. CNCs are commonly employed in the formulation of moisturizers, modifiers, and additives, playing a key role in the creation of bioactive carrier compounds, skin sensors, and UV blockers (Rashid & Dutta 2020). For instance, hemp-derived CNC grafted onto PLA has been utilized in skin protection products, demonstrating excellent water resistance, biocompatibility, and non-penetration into the skin (Zhao et al. 2020). The hemp/CNC-g-PLA aqua-based foundation retains beneficial characteristics such as reducing skin roughness, being easy to remove, and addressing discoloration and age spots, making it valuable for protecting skin from excessive cleansing damage (Tang et al. 2021). Additionally, CNC excels in cosmetic applications as an emulsion stabilizer and enzyme immobilizer, contributing to skin rejuvenation by combating wrinkles, dark spots, dehydration, and photoaging (Tang et al. 2021).

### Electronics

CNC materials are valued for their ability to contribute to cost-effective and efficient electronic components. Their unique properties include a high surface-to-volume ratio, eco-friendliness, lightweight nature, porosity, hydrogen-bonding capacity, mechanical strength, ease of surface modification, and thermal stability. Thanks to these properties, CNCs are increasingly being incorporated into electroactive materials, allowing for the creation of flexible and conductive electronic components.

Researchers have engineered CNCs from carbon sources to produce strong electrically conductive materials. These materials are used as electron collectors in energy storage batteries, sensors, and high-capacity energy devices such as supercapacitors (Al Haj et al. 2022; Nang An et al. 2020; Shahi et al. 2021; Taer et al. 2019). The porous structure of CNCs enhances electron and ion transfer, making them highly effective in supercapacitors that incorporate conductive materials. CNCs are also being widely used in various other electronic applications, including soft actuators, multipurpose electronics, sensitive hydrogels, optical filters, and anti-reflective coatings (Mateo et al. 2021). These advances highlight the growing role of CNCs in developing more efficient and flexible electronic components for next-generation technologies.

### Paints and coatings

CNC also plays a crucial role in the manufacturing of paints and coatings, where it enhances both durability and performance. The incorporation of CNC into paints has been shown to improve key properties such as color brightness, smoothness, color fastness, tolerance, and mechanical strength (Rajinipriya et al. 2018). In addition, when CNC is added to polymeric matrices for edible coatings, it improves barrier and mechanical properties while stabilizing the release of active agents, helping to extend the shelf life of products without compromising quality (Pirozzi et al. 2021). Furthermore, applying a CNC layer onto electrospun polyamide 6 (PA6) films has been found to reduce porosity while increasing mechanical strength and surface roughness (Tan et al. 2022). These enhancements make CNC a valuable addition to the formulation of advanced paints and coatings, contributing to improved aesthetics and performance.

## Conclusion and future recommendations

This study provides an in-depth analysis of CNC synthesis methods, properties, and industrial applications, with a focus on agricultural and forestry biomass as primary raw materials. Proximate and elemental analyses, along with the lignocellulosic composition of these biomass sources, are essential for determining CNC yield and properties. High cellulose and carbon content, as well as increased volatile matter, generally enhance CNC yield, while elevated lignin content, moisture, and oxygen negatively hinder the production process.

The CNC synthesis process involves several key stages: pretreatment steps such as cleaning, washing, drying, and size reduction, followed by alkali treatment for delignification, bleaching, and hydrolysis through various methods. These include acid, mineral, organic acid, solid acid, ionic liquid, and enzymatic approaches. Additionally, this study highlights CNC’s chemical (FTIR analysis), structural (SEM/FESEM, TEM, XRD), and thermal (TGA/DTG, DSC) properties, as well as its particle size, particularly from diverse biomass sources.

CNC has broad industrial applications, spanning sectors such as wastewater treatment, food packaging, polymer nanocomposites, biomedicine, pharmacy, cosmetics, paints and coatings, and electronics. With its combination of strong mechanical properties, thermal stability, biodegradability, and ease of surface modification, CNC stands out as a valuable material in many industries. However, despite its vast potential, research on CNC is still in its early stages, particularly regarding commercial scalability and broader applications.

To address these challenges, future research should focus on several critical areas. One major challenge is the development of more efficient methods for large-scale CNC production from lignocellulosic resources. Optimizing raw materials rich in cellulose and low in hemicellulose and lignin will be key to improving CNC yield and performance in new applications. The current multi-step CNC synthesis processes are time-consuming and commercially inefficient, increasing production costs. Simplifying these methods is crucial for enhancing CNC’s commercial viability. Moreover, the high consumption of costly chemicals and the limited recyclability of these materials raise environmental concerns due to the production of toxic by-products. Future research should explore strategies to reduce chemical usage and enhance the recyclability of expensive reagents. The development of efficient catalysts to minimize acid consumption and improve process efficiency is also a critical area of focus.

Optimizing the overall process, including pretreatment and hydrolysis, is key to achieving higher yields with improved properties. This will expand CNC’s applications across a wider range of industries. Surface modification presents further opportunities, as it could enhance CNC’s properties and enable new industrial uses. Proper characterization of CNC before and after surface modification is essential to fully understand its capabilities. While significant progress has been made, several challenges remain in CNC synthesis, property enhancement, and industrial applications. CNC preparation can be labor-intensive, and equipment corrosion during production is a recurring issue. Additionally, there is untapped potential for CNC in emerging industries, including energy, sensors, supercapacitors, bioengineering, tissue engineering, 3D printing, bioremediation, biofuels, and flame-retardant materials. Future studies should prioritize the commercial scalability of CNC production and explore its broader application across industries, fostering continued innovation in material science and sustainability.

## Supplementary Information

Below is the link to the electronic supplementary material.

Supplementary file1 (DOCX 34 KB)

## Data Availability

Not applicable (for this review paper).
