# Supplementary material for: Cellulose nanocrystals from agriculture and forestry biomass: synthesis methods, characterization and industrial applications
Source: Environ Sci Pollut Res Int. 2024 Sep 28;31(49):58745–78. doi: 10.1007/s11356-024-35127-3 (PMC11513767; doi:10.1007/s11356-024-35127-3)
Supplement: Supplementary file 1 — Supplementary file1 (DOCX 34 KB) [file 11356_2024_35127_MOESM1_ESM.docx]

**Figure: S1.** Global distribution of agricultural areas over the continents
